# Transcriptome Analysis of Responses to Dengue Virus 2 Infection in *Aedes albopictus* (Skuse) C6/36 Cells

**DOI:** 10.3390/v13020343

**Published:** 2021-02-22

**Authors:** Manjin Li, Dan Xing, Duo Su, Di Wang, Heting Gao, Cejie Lan, Zhenyu Gu, Tongyan Zhao, Chunxiao Li

**Affiliations:** State Key Laboratory of Pathogen and Biosecurity, Beijing Institute of Microbiology and Epidemiology, Beijing 100071, China; lmj18325391737@126.com (M.L.); xingdan93@163.com (D.X.); duosu@hotmail.com (D.S.); m17806280983@163.com (D.W.); gaoheting1006@163.com (H.G.); lancejie@126.com (C.L.); 18810500283@163.com (Z.G.); tongyanzhao@126.com (T.Z.)

**Keywords:** *Aedes albopictus*, C6/36 cell, DENV2, RNA-Seq, transcriptome

## Abstract

Dengue virus (DENV), a member of the *Flavivirus* genus of the Flaviviridae family, can cause dengue fever (DF) and more serious diseases and thus imposes a heavy burden worldwide. As the main vector of DENV, mosquitoes are a serious hazard. After infection, they induce a complex host–pathogen interaction mechanism. Our goal is to further study the interaction mechanism of viruses in homologous, sensitive, and repeatable C6/36 cell vectors. Transcriptome sequencing (RNA-Seq) technology was applied to the host transcript profiles of C6/36 cells infected with DENV2. Then, bioinformatics analysis was used to identify significant differentially expressed genes and the associated biological processes. Quantitative reverse transcription-polymerase chain reaction (qRT-PCR) was performed to verify the sequencing data. A total of 1239 DEGs were found by transcriptional analysis of *Aedes albopictus* C6/36 cells that were infected and uninfected with dengue virus, among which 1133 were upregulated and 106 were downregulated. Further bioinformatics analysis showed that the upregulated DEGs were significantly enriched in signaling pathways such as the MAPK, Hippo, FoxO, Wnt, mTOR, and Notch; metabolic pathways and cellular physiological processes such as autophagy, endocytosis, and apoptosis. Downregulated DEGs were mainly enriched in DNA replication, pyrimidine metabolism, and repair pathways, including BER, NER, and MMR. The qRT-PCR results showed that the concordance between the RNA-Seq and RT-qPCR data was very high (92.3%). The results of this study provide more information about DENV2 infection of C6/36 cells at the transcriptome level, laying a foundation for further research on mosquito vector–virus interactions. These data provide candidate antiviral genes that can be used for further functional verification in the future.

## 1. Introduction

A member of the *Flavivirus* genus of the Flaviviridae family, the dengue virus is of great significance worldwide, causing pandemics that infect millions of people in more than 100 countries every year. Currently, there are no effective vaccines or antiviral drugs, and the prevention and treatment of infection with this virus is more difficult than those of other viral infections because the risk of secondary infection is caused by enhanced antibody-mediated immunity [1,2]. Vector control, the most effective strategy for preventing transmission, needs to be improved. DENV is mainly transmitted by aedes mosquitoes. By ingesting viral blood meal, the midgut tissue of mosquitoes is first infected, then spreads to other tissues through hemolymph, such as trachea, fat body, and salivary glands, and enters saliva through replication during transmission. Arbovirus infection usually causes pathological symptoms in the human body, and the vector usually does not produce pathological changes or is relatively mild. At present, people do not know much about the mechanism of virus infection and transmission in insect, and the virus defense system is even worse.

In arthropods, innate immunity plays an important role in inhibiting pathogen infection through the production of effector molecules such as antimicrobial peptides, physical barriers such as phagocytosis, embedding, and secretion, and melanization [3]. Studies have shown that some classic innate immune pathways, such as RNAi, Toll, and JAK-STAT pathways, are involved in inhibiting viral infection and related defense mechanisms in mosquitoes [4,5,6,7]. Despite the above research results, the molecular mechanisms that regulate activation after infection and other regulatory mechanisms related to viral infection such as phagocytosis, autophagy, apoptosis, metabolism, and proteolysis are still unclear [8]. In recent years, people have become interested in the field of virus–mosquito interactions. Research on these interactions requires many special skills and equipment, including mosquito capture and breeding, as well as special methods and expertise in handling mosquito vectors. Moreover, the regulation of viral infections in mosquitoes is complex, and different tissues and body parts are different. Therefore, a simple and operable alternative model is needed to study vector–pathogen interactions. A cell line derived from the mosquito host provides a relatively simple tool for research due to their homologous, sensitive, and repeatable, and is currently an important platform for studying insect biology and virology [9].

The C6/36 cell line was established in 1967 from *Aedes albopictus* larvae [10]. The C6/36 cell line is often used for arbovirus detection, propagation, and other research [11]. Its population doubling time is very short, and it can be easily infected by mosquito-borne viruses that are members of the Flaviviridae, Bunyaviridae, and Togaviridae families, partially due to the lack of a functional RNAi response [12]. The subclone C6/36 was chosen because of its high virus production and showed that it retains a diploid karyotype with 2n = 6 chromosomes in most cells [13]. It is the most commonly used cell line to study the contents of mosquito-borne virus infection. For example, a defective DENV genome was detected in C6/36 cells, which helped facilitate further understanding of the mechanism of arbovirus infection [14]. Furthermore, C6/36 cells have been used to investigate the invasion mechanism of DENV in some studies [15].

To date, little is known about the mechanism of C6/36 cell infection with DENV2. Recently, RNA-Seq has been recognized as an effective transcriptome analysis method that can analyze gene expression and identify new RNA copies, and it is superior to traditional methods in terms of accuracy, repeatability, and coverage [16,17]. Transcriptome sequencing (RNA-Seq) analysis of C6/36 host cell transcript profiles with or without DENV2 infection showed that DENV2 infection significantly altered the transcription of C6/36 cells. C6/36 assembly was compared with the *Ae. albopictus* genome [18]. The RNA-Seq data have been stored in public databases to allow further study of DENV2 and other viruses circulating in *Ae. albopictus* and C6/36 cell lines.

## 2. Materials and Methods

### 2.1. Cell Culture and Denv2 Strain

*Ae. albopictus* C6/36 cells were cultured in RPMI 1640 basic medium (RPMI; Gibco, Grand Island, NY, USA) comprising 10% fetal bovine serum (FBS; Gibco, Grand Island, NY, USA) at 28 °C in 5% CO_2_. BHK-21 cells were used to determine the virus titer, which were cultured in Dulbecco’s modified Eagle’s medium (DMEM; Gibco, Grand Island, NY, USA) comprising 10% FBS, at 37 °C in 5% CO2. The DENV2 Guangdong strain, which was provided by Guangdong Provincial Centers for Disease Control and Prevention, was subcultured from the brains of suckling mice. The virus titer was determined by a BHK-21 monolayer plaque formation assay.

### 2.2. Virus Infection

First, 5 mL of C6/36 cell suspension was seeded in a 25 cm^2^ cell culture flask (1 × 10^6^ cells/mL). After 24 h of culture, 1 mL of DENV2 dilution was added to each cell culture flask to make the working concentration of DENV2 reach a multiplicity of infection (MOI) of 1. Then, the cells were placed in the incubator and gently shaken every 15 min to make the infection uniform, and this process was 4 times. The viral suspension was aspirated, and 5 mL of RPMI medium comprising 0.1% antibiotic and 2% FBS was added. Morphological changes in C6/36 cells were observed at 0, 1, 2, 3, 4, 5, 6, 7, and 8 days post infection (dpi). Viral copy number detection was performed using the DENV2 nucleic acid extraction-free detection kit (Suneye Biotechnology, Beijing, China). To confirm the production of infectious virions from C6/36 cells, plaque assays were used to analyze the infected cell samples [19].

### 2.3. cDNA Library Construction and Transcriptome Sequencing

Infected cells and uninfected cells at 4 dpi were sampled for transcriptome sequencing, with three biological replicates in each group. A cDNA library was prepared using the NEBNext^®^ ultraTM RNA Library Prep Kit for Illumina^®^ (New England Biolabs, Beverly, MA, USA). Upon completion, the library was preliminarily quantified using a Qubit2.0 fluorometer (Thermo Fisher Scientific, Waltham, MA, USA) and diluted to 1.5 ng/µL, and then an Agilent 2100 bioanalyzer (Agilent Technologies, Santa Clara, CA, USA) was used to determine the insert size of the library. After it was confirmed that the insert size was as expected, qRT-PCR was used to accurately quantify the effective concentration of the library (above 2 nM) to ensure the quality of the library. Illumina sequencing was performed after pooling different libraries according to the effective concentration and the target data volume. The basic principle of sequencing was sequencing by synthesis. An Illumina NovaSeq 6000 sequencer (Illumina, San Diego, CA, USA) was used for sequencing. When each sequencing cluster extended the complementary chain, the sequencer captured the fluorescence signal of dNTPs with fluorescent labels, and the optical signal was converted into the sequencing peak with computer software to obtain the sequence information of the fragment to be tested.

### 2.4. Sequencing Analyses

To ensure the quality and reliability of data analysis, it was necessary to filter the original data. Reads with adapters, undetermined base information, and low quality were removed. Clean reads were quickly and accurately compared with the reference genome using Hisat2 v2.0.5 to obtain the localization information of reads on the reference genome [20]. The Integrative Genomics Viewer (IGV) was used for visual browsing in combination with the species reference genome and annotation files. The new transcripts were assembled by StringTie (v1.3.3b) in a novel network flow algorithm as well as with an optional de novo assembly approach [21]. The number of reads covered from start to finish for each gene (including the new predictive gene) was counted based on the location information of gene alignment on the reference genome. This part of the analysis uses FeatureCounts v1.5.0-p3 [22]. The obtained read count value was then corrected for sequencing depth and gene length to obtain FPKM values (FPKM, the expected number of fragments per kilobase of transcript sequence per million base pairs sequenced). Considering the biological duplication in this experiment, the DESeq2 R package was used for differential analysis [23,24]. The screening criteria for differentially expressed genes (DEGs) were padj < 0.05 and |Log2 (FC)| > 1 (fold change). Then, the clusterProfiler R Package was used for gene ontology (GO) and pathway enrichment analysis [25].

### 2.5. qRT-PCR Validation of RNA-Seq Data

To confirm the RNA-Seq data, we verified the differential expression of some mRNA transcripts by qRT-PCR. Table 1 shows the primer sequences of selected mRNA transcripts and the reference gene β-actin [26]. In this experiment, there were 4 biological replicates in each group at 1, 2, and 4 dpi. Four biological replicates were obtained from the infected group and uninfected group. The qRT-PCR experiment used the PerfectStart™ Green qPCR SuperMix Kit (Transgene, Beijing, China). The reaction system consisted of 2.0 µL of cDNA template, 10 µL of SuperMix, 0.4 µL of Passive Reference Dye II, 6.8 µL of RNase-free H_2_O, and 0.4 µL of 10 mM F/R primer (BGI, Shenzhen, China). qPCR was performed using the ABI QuantStudio 7 flex Real-Time PCR system (Thermo Fisher Scientific, Waltham, MA, USA). The reaction procedure was as follows: 94 °C for 30 s for 1 cycle; 94 °C for 5 s and 60 °C for 30 s for 40 cycles; and dissociation stage. The 2^−ΔΔCT^ method was used to analyze the qRT-PCR results, and the reference gene was β-actin [27,28].

## 3. Results

### 3.1. Infection of C6/36 Cells with DENV2

**Figure 1:**
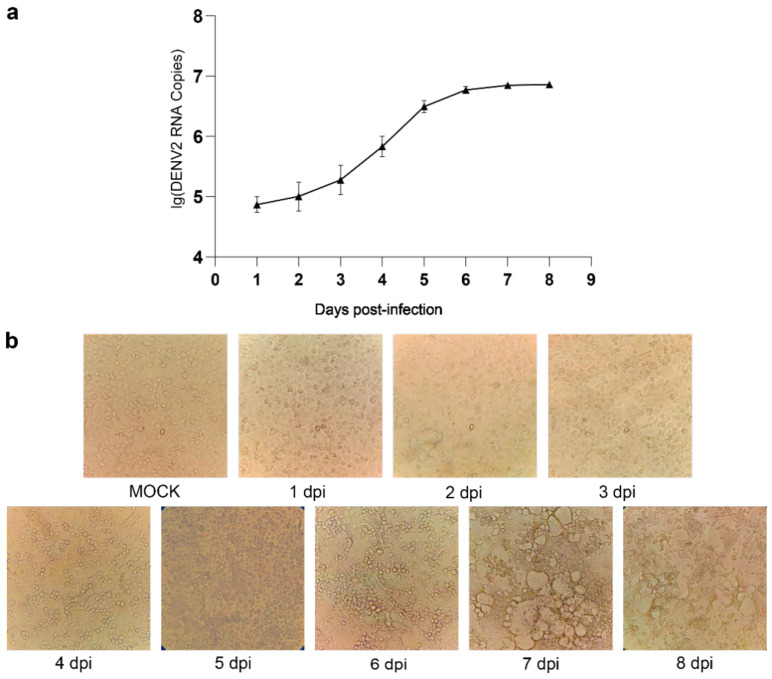
Viral growth kinetics of (**a**) and morphological changes in (**b**) C6/36 cells infected with DENV2. (**a**) The *Y*-axis label represents log10 (DENV2 RNA copies), and the *X*-axis label represents days post-infection with DENV2. (**b**) Mock refers to uninfected C6/36 cells (dpi: days post-infection). The magnification is 200 ×.

We investigated the host cell responses of C6/36 cells to DENV2 infection. To evaluate growth kinetics, the supernatants of C6/36 cells infected with DENV2 were harvested at 0, 1, 2, 3, 4, 5, 6, 7, and 8 dpi, and the virus copy number at each time point was determined via qRT-PCR (Figure 1a). The results indicated that the virus copy number gradually increased, reaching a peak at 6 to 7 dpi and then plateauing. Cytopathic effect (CPE) or morphological changes were observed in DENV2-infected C6/36 cells but not mock-infected cells at 4 dpi, with the infect cells gradually showing obvious phenotypes such as cavitation, collapse, and fusion, which is different from the results in Aag2 cells found in our previous research, which showed no changes in morphology or CPE following DENV2 infection (Figure 1b) [29]. DENV2- and mock-infected cells were harvested at 4 dpi for transcriptome sequencing analysis. To quantify the production of infectious virions by C6/36 cells, viral supernatants and cells were assayed for infectious virus at 4 dpi, and the virus titers were 7.0 × 10^5^ and 1.0 × 10^3^ PFU/mL, respectively.

### 3.2. RNA-Seq Data and DEG Analysis

**Figure 2:**
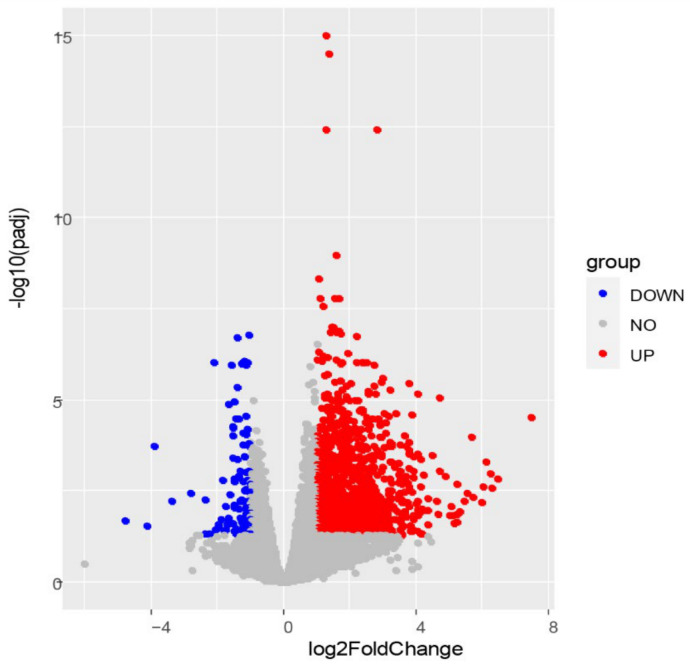
Volcano plot of differentially expressed genes. The red dots represent the 1133 upregulated genes; the blue dots represent the 106 downregulated genes; the gray dots represent the unchanged genes.

To analyze the transcriptome changes in C6/36 cells induced by DENV2 infection, cell samples were subjected to sequencing at 4 dpi. There were three biological replicates in the infected group and the uninfected group. The infection group included 46,685,278, 46,976,324, and 47,320,364 raw reads, and the control group included 47,587,424, 45,682,174, and 46,236,094 raw reads. After data filtering, there were 45,886,806, 46,102,854, and 46,642,230 clear reads in the infected group, while 46,569,518, 44,845,032, and 45,348,116 were obtained from the control group. After the new transcripts were assembled, they were annotated with the Pfam database, and a total of 1833 new mRNA transcripts were predicted. Differentially expressed genes were identified using DESeq2 and are shown in volcano plots (Figure 2). A padj < 0.05 and |Log2 (FC)| > 1 were taken as the thresholds, and 1239 DEGs that may be associated with DENV2 infection were identified (Table S1). Among them, 1133 genes were upregulated, with log2 (FC) values ranging from 1.00 to 7.49. In addition, 106 genes were downregulated, with log2 (FC) values ranging from −4.77 to −1.00.

### 3.3. GO Enrichment Analysis of DEGs

**Figure 3:**
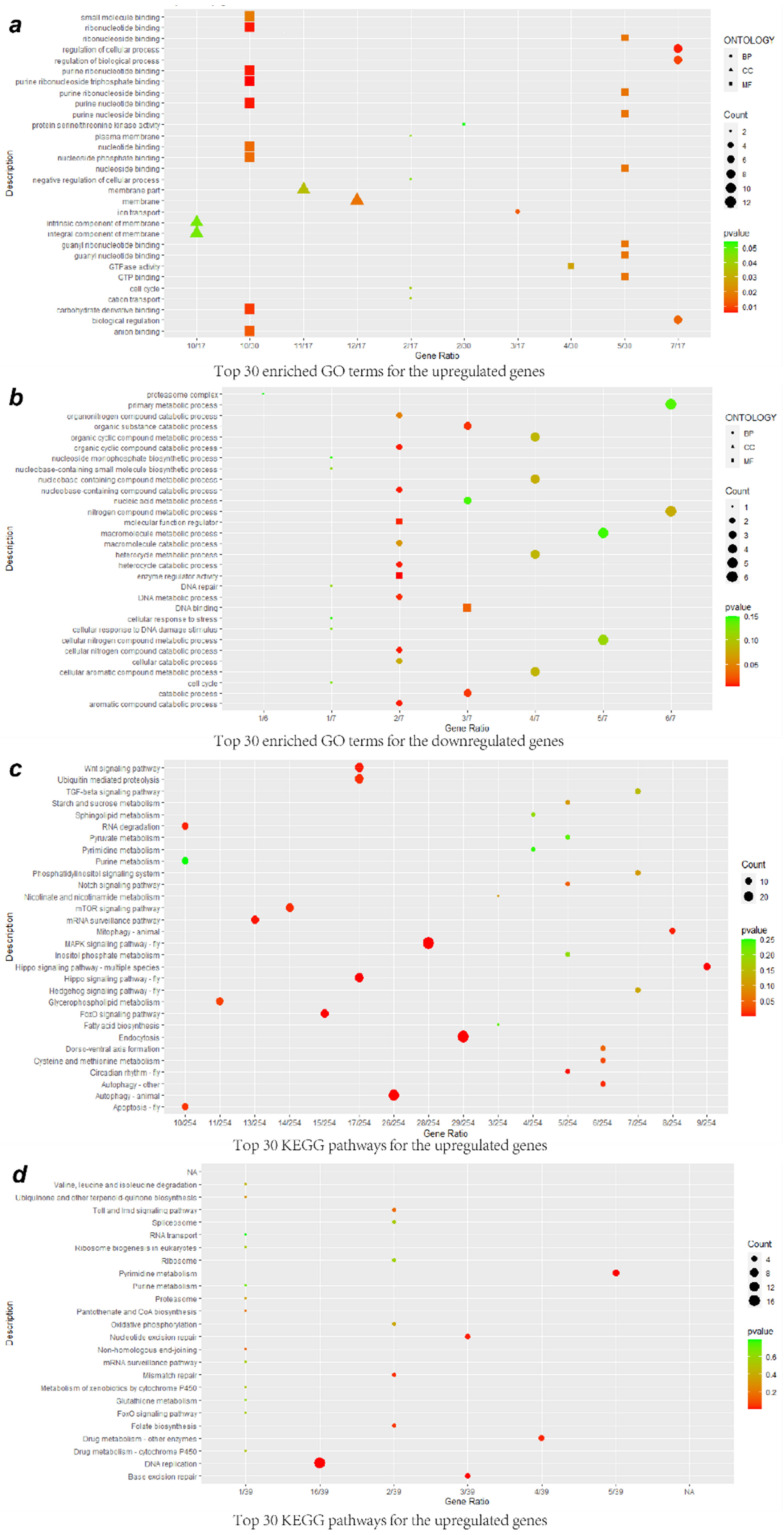
Bubble plots comparing GO enrichment and KEGG pathway analysis. (**a**) The 30 most significantly enriched GO terms for upregulated DEGs. (**b**) The 30 most significantly enriched GO terms for downregulated DEGs. (**c**) The 30 most significantly enriched KEGG pathways for upregulated DEGs. (**d**) The 30 most significantly enriched KEGG pathways for downregulated DEGs. The size of the bubble represents the number of enriched DEGs. The color represents the *p*-value of the enrichment. The shape of the bubble represents the ontology; circles represent BP, triangles represent CC, and squares represent MF.

To further analyze the relevant biological functions of DEGs, the upregulated DEGs were subjected to GO enrichment analysis, and 222 terms were obtained, including 125 biological process (BP), 41 cellular component (CC), and 56 molecular function (MF) terms (Figure 3a; Table S2). Regarding the BP terms, the upregulated DEGs were significantly enriched in the regulation of cellular process and biological process, ion transport, biological regulation, cation transport, cell cycle, and negative regulation of cellular process; the CC terms mainly included membrane, membrane part, integral component of membrane, and intrinsic component of membrane; and regarding the MF terms, the categories most enriched were material binding and enzyme activity, such as those involving purine ribonucleoside triphosphate binding, ribonucleotide binding, carbohydrate derivative binding, anion binding, GTP binding, small molecule binding, and GTPase activity, among others. The downregulated DEGs were associated with 156 terms, including 85 BP terms, 35 CC terms, and 36 MF terms (Figure 3b; Table S3). In the BP category, the downregulated DEGs were involved in catabolic processes, including processes involving aromatic compounds, nucleobase-containing compounds, cellular nitrogen compounds, heterocycles, organic cyclic compounds, organic substances, and organonitrogen compounds, metabolism of DNA, nitrogen compounds, and nucleobase-containing compounds, among other processes. For the CC category, the DEGs were found to participate in proteasome complexes, endopeptidase complexes, nonmembrane-bound organelles, intracellular nonmembrane-bound organelles, and nuclei. Among the MF terms, enzyme regulator activity, molecular function regulator, and DNA binding were the most highly represented categories.

### 3.4. KEGG Enrichment Analysis of DEGs

Furthermore, all DEGs were functionally classified according to Kyoto Encyclopedia of Genes and Genomics (KEGG) pathway analysis. The upregulated DEGs were distributed into 82 KEGG pathways (Figure 3c; Table S4). The highly abundant KEGG pathways included several signaling pathways, such as the MAPK, Hippo, FoxO, Wnt, mTOR, and Notch pathways; metabolic pathways of glycerophospholipid and cysteine and methionine; cellular physiological processes, such as autophagy, endocytosis, and apoptosis; and other processes, such as circadian rhythm, mRNA surveillance pathway, mitophagy, RNA degradation, ubiquitin-mediated proteolysis, and dorsoventral axis formation. The downregulated DEGs participated in a total of 24 pathways, including DNA replication, pyrimidine metabolism, base excision repair, nucleotide excision repair, drug metabolism—other enzymes, mismatch repair, and folate biosynthesis (Figure 3d; Table S5). Furthermore, the upregulated and downregulated DEGs were simultaneously enriched in the Toll and Imd signaling pathways (ID: aalb04624, up: 6, down: 2), which are involved in multiple processes and immune responses.

### 3.5. RNA-Seq Data Validation by qRT-PCR

**Figure 4:**
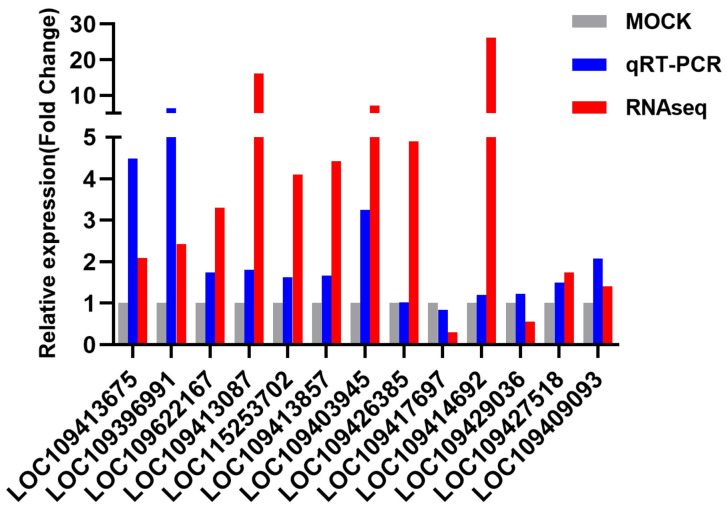
qRT-PCR and transcriptome analysis of 13 differentially expressed genes. Mock means uninfected cells, and the FC values of these cells were set to 1. The relative expression level (FC) of a mRNA transcript refers to the change in expression of DENV2-infected C6/36 cells relative to uninfected cells determined by using the 2^−ΔΔCT^ method.

The relative expression results showed that the FC was between 0.84 and 6.41 (Figure 4). Among the 13 analyzed genes, the expression trends of 12 genes were consistent, and the trend of 1 gene was inconsistent. There was a high consistency between the RNA-Seq and RT-qPCR data (92.3%). Moreover, the expression levels of 8 genes were significantly upregulated (*p* < 0.05). The IDs of these genes are LOC109413675, LOC109396991, LOC109622167, LOC109413087, LOC109413857, LOC109403945, LOC109427518, and LOC109409093, and the FC range was 1.50–6.41. Furthermore, to assess the physiological development processes of these 13 genes in C6/36 cells, we also verified their relative expression at 1 and 2 dpi (Table 2). At 1 dpi, the FC range was 0.03–1.89, and the expression of LOC109396991 and LOC109429036 was significantly upregulated (*p* < 0.05). At 2 dpi, the FC value was between 0.55 and 1.85, and the expression of LOC109396991 and LOC109429036 was significantly upregulated (*p* < 0.05), while the expression of LOC109426385, LOC109417697, and LOC109427518 was significantly downregulated (*p* < 0.05).

### 3.6. Comparison between C6/36 Cell and Aag2 Cell Transcriptomes

**Figure 5:**
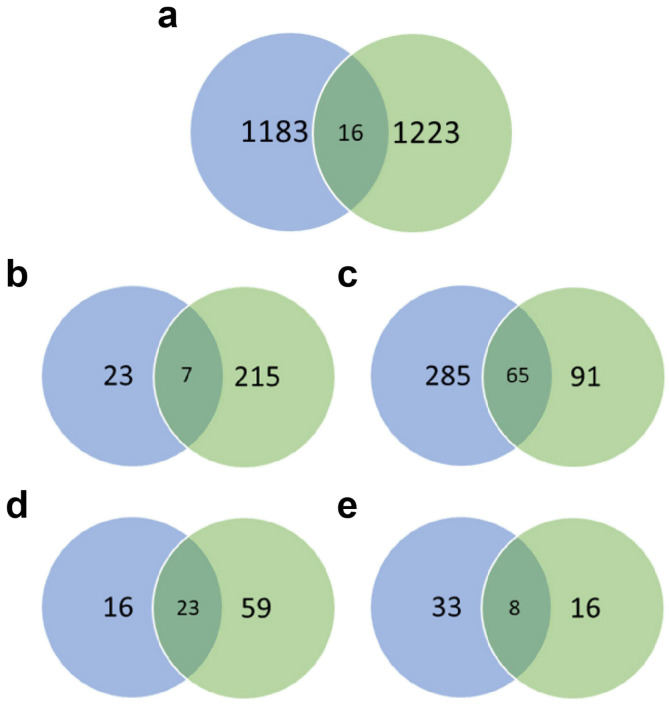
Overlap analysis of the transcriptomes of C6/36 and Aag2 cells. Blue represents the transcriptome analysis results of Aag2 cells, and green represents the transcriptome analysis results of C6/36 cells. (**a**) The 16 overlapping DEGs between the Aag2 and C6/36 cell lines. (**b**) The 7 overlapping GO terms enriched for upregulated DEGs. (**c**) The 65 overlapping GO terms for downregulated DEGs. (**d**) The 23 overlapping KEGG pathways enriched for upregulated DEGs. (**e**) The 8 overlapping KEGG pathways for downregulated DEGs.

In our previous research, we also performed transcriptome sequencing on 4 dpi-Aag2 cells to analyze and verify the transcriptome changes induced by DENV2 infection. In these two experiments, we used the same DENV2 strain (DENV2 Guangdong strain, MOI is 1), and both samples were taken on the 4 dpi for transcriptome sequencing. The sequencing was completed in the same company. The subsequent bioinformatics analysis methods used are the same, and the screening criteria for differentially expressed genes used are the same (padj < 0.05 and |Log2 (FC)| > 1). A total of 16 DEGs overlapped between Aag2 cells (which we studied before [29]) and C6/36 cells (Figure 5a). GO enrichment analysis revealed 7 overlapping terms enriched for upregulated DEGs (Figure 5b), which mainly included phosphate-containing compound and phosphorus metabolic process, biosynthetic processes such as nucleobase-containing compound, aromatic compound, heterocycle, organic cyclic compound, and DNA binding. Sixty-five downregulated DEGs (Figure 5c) were mainly involved in catabolic processes, including aromatic compounds, nucleobase-containing compounds, cellular nitrogen compounds, heterocycles, organic cyclic compounds, and organic substances. According to the KEGG enrichment results, 23 overlapping pathways were enriched for upregulated DEGs (Figure 5d), including pyrimidine metabolism, longevity regulating pathway, drug metabolism, starch and sucrose metabolism, FoxO signaling pathway, and circadian rhythm. Eight downregulated DEGs were involved in drug metabolism (Figure 5e), folate biosynthesis, Toll and Imd signaling pathway, valine, leucine and isoleucine degradation, spliceosome, mRNA surveillance pathway, purine metabolism, and RNA transport.

Three genes of homologous targets among the 13 genes validated by qRT-PCR were also verified to be differentially expressed in Aag2 cells (UDP-glucuronosyltransferase 2B1-like, LOC109429036; cystathionine beta-synthase, LOC109427518; and facilitated trehalose transporter Tret1, LOC109409093). Unlike in C6/36 cells (for which gene expression was analyzed by qRT-PCR), the gene expression of these three genes was downregulated at 4 dpi in Aag2 cells. The expression of the homologs of LOC109429036 and LOC109427518 was significantly downregulated in Aag2 cells (*p* < 0.05).

## 4. Discussion

Dengue virus can cause diseases such as dengue fever (DF) and even more severe conditions such as dengue hemorrhagic fever (DHF) and dengue shock syndrome (DSS). [30]. Humans are the only hosts of epidemic DENV strains, which develop complex mechanisms to evade human innate immune responses [31]. *Aedes mosquitoes* are the vectors through which viruses can replicate and spread. In this study, we used RNA-Seq technology to study the transcriptional changes in DENV2-infected and uninfected *Ae. Albopictus* C6/36 cells. Previous transcriptome studies used whole mosquitoes [32,33,34,35,36], and our goal was to further study the interaction with viruses in homologous, sensitive, and replicable cell vectors. In this study, to avoid the effects of RNA degradation and accumulation on physiological functions and to ensure the quality of the cDNA library, the selected samples were collected at four dpi that exhibited high virus titers and copy numbers after DENV2 infection for transcriptome sequencing. Moreover, CPE began to be observed in DENV2-infected C6/36 cells at 4 dpi. By the 6 dpi, most of the cells have been completely broken, so there is no value to study their physiological functions. Therefore, here we mainly want to explore the transcriptomic changes at a representative time point when significant morphological changes begin to occur during the physiological process of C6/36 cell virus infection.

A total of 1239 DEGs were identified in this experiment, among which 1133 genes were upregulated and 106 genes were downregulated. Analysis of gene expression patterns revealed possible transcriptional changes in virus-infected cells compared with uninfected cell. Enrichment analyses were also conducted to provide insight into the functions of the DEGs in viral infection, understand the key biological processes in which they are involved, and reveal their basic molecular mechanisms. According to the enrichment results, most DEGs were related to signaling, metabolism, repair pathways, and some cellular physiological processes involving autophagy, endocytosis, and apoptosis.

Similar to the results of our previous research in Aag2 cells [29], cells maintain a dynamic balance by changing their metabolic pathways during the long-term evolution process to ensure their survival after infection [37]. Among these processes, pyrimidine metabolism, which we also observed to be enriched in this study, has been proven to affect virus replication and pathogen infection [38,39]. In addition, the DEGs were enriched in glycerophospholipid metabolic, cysteine and methionine metabolic pathways. In many other virus-related studies, it was found that the glycerophospholipid metabolic pathway is strongly related to virus replication, and an increase in glycerophospholipid is consistent with the kinetics of virus replication [40,41]. The mechanism underlying how glycerophospholipid metabolism participates in virus replication needs further study.

The MAPK, Hippo, FoxO, mTOR, and Notch signaling pathways are mainly involved in the regulation of cell metabolism, differentiation, growth, proliferation, survival, apoptosis, and death and other various physiological processes. Most signaling pathways participate in the regulation of virus infection by participating in apoptosis, also known as programmed cell death (PCD). As an important defense mechanism by which the host resists pathogen invasion, apoptosis adjusts the dynamic balance of physiological processes [42]. The virus is recognized on the surface of the cell by activating the signal transduction pathway so that the cell undergoes an endocytosis reaction for entry, generates early cytoplasmic events, and optimizes the replication cycle [43].

Endocytosis provides an effective method for viruses to infect host cells by mediating virus internalization and transport to replication sites [44,45]. DENV2 enters insect cells through receptor-mediated clathrin-dependent endocytosis and needs to be transported through an acidic pH compartment to subsequently uncoat and complete productive infection [46]. In our study, some upregulated DEGs were involved in endocytosis, which once again provides proof of how DENV2 enters C6/36 cells. However, it is not yet clear how differentially expressed genes are involved in the endocytic pathway of C6/36 cell infection, and further research is needed. However, from this perspective, some inhibitors of clathrin-mediated endocytosis can be used to inhibit viral replication, including small molecules (monodansylcadaverine and chlorpromazine), osmotics (hypertonic sucrose), dominant interfering mutant proteins (dynamin), and loss-of-function reagents (siRNAs). Studies have shown that the use of these inhibitors can inhibit DENV2 virus yield and protein expression. Moreover, it provides new ideas and methods to reduce the amount of virus by preventing the entry of the virus.

Autophagy is a normal process for the natural attrition and renewal of organelles and other structures in cells. When cells are damaged by various physical and chemical factors, autophagic lysosomes increase in number, has allowing them to have a protective effect against cell damage [47]. Autophagy has been recognized as an innate immune response that can lead to the death of pathogen-infected cells [31]. Studies have found that some positive-strand RNA viruses can promote the production of degradative autolysosomes, thereby promoting their replication, including hepatitis C virus (HCV), poliovirus (PV), and DENV2 [48]. In this study, it was also found that DENV2 infection activated the autophagy pathway. Studies have shown that the activation of the autophagy pathway in turn increases the virus titer and have identified ATG5 as the key gene involved in this process [49]. This indicates that autophagy can promote virus replication in infected cells. In addition, studies have used the inhibitor spautin-1 to inhibit autophagy and only produce noninfectious dengue virus particles. It is believed that the production of infectious virus particles requires autophagy [50]. The use of drug inhibitors is a good antiviral strategy, and autophagy inhibitors may prove to protect against anti-dengue virus infection and act as effective therapeutic agents.

The mRNA surveillance pathway is a control mechanism for assessing the quality of mRNA, which can detect and degrade abnormal mRNA and prevent the synthesis of potentially toxic proteins. This pathway includes nonsense-mediated decay (NMD), nonstop decay (NSD), and no-go decay (NGD) [51]. The NMD pathway is mostly studied in research on viruses. In previous studies, coronavirus (CoV) and Zika virus (ZIKV) infections were shown to inhibit or disrupt the NMD pathway, and the NMD pathway degrades viral mRNA in the early stages of infection, thereby interfering with viral replication [52,53]. However, our data show that DENV2 virus infection promotes this pathway. The study also found that the inhibition of NMD regulator up-frameshift protein 1 (UPF1) enhanced ZIKV infection. Our research provides basic information about the biological importance of the mRNA surveillance pathway in mRNA quality monitoring and virus replication efficiency. The ubiquitin-mediated proteolytic pathway, which we also showed was enriched, participates in the degradation of native cellular proteins [54]. It is involved in some cellular physiological processes, including the cell cycle and division, cellular stress response, apoptosis, immune response, DNA repair, transcription regulation, and signal transduction [55].

The circadian rhythm pathway was enriched in Aag2 and C6/36 cells infected with DENV2. As we explained in previous studies [29], the molecular mechanism of this pathway is not fully understood, but many studies have fully demonstrated the impact of circadian rhythm disruption on infections and diseases. In addition, the upregulated and downregulated DEGs were simultaneously enriched in the Toll and Imd signaling pathways, but the JAK-STAT signaling pathway was not identified, which is similar to the results of our previous analysis of the transcriptome of DENV2-infected Aag2 cells [29]. These findings strongly suggest that the Toll and Imd signaling pathways may participate in the regulation of DENV2–vector interactions. It was once again confirmed that the Toll and JAK-STAT signaling pathways act in two distinct antiviral networks.

The downregulated genes were mainly enriched in certain repair pathways, such as base excision repair (BER), nucleotide excision repair (NER), and mismatch repair (MMR). Over the course of long-term evolution, the virus developed a mechanism that inhibits host DNA repair, thereby circumventing its detection, and uses this mechanism to drive its own replication. Many studies have found interactions between DNA viruses and cellular DNA damage repair pathways [56]. In addition, viral proteins can directly interact with cell components to regulate the cell cycle or replication stress, thereby affecting sensing and repair mechanisms. Many viruses participate in the regulation of DNA damage signal transduction and repair pathways during the replication process. Our data show that C6/36 cells infected with DENV2 may produce a substantial amount of DNA damage, and DENV2 infection and replication inhibit DNA repair pathways, especially BER, NER, and MMR. Studies have also found that infection with many RNA viruses can disrupt the DNA repair system, including influenza A virus (IAV) and hepatitis C virus (HCV), and MMR plays an important role in cell survival [57,58]. Our research may reveal the molecular mechanism of C6/36 cell infection induced by DENV2 from another perspective, which may be related to the morphological changes in C6/36 cells. As the main regulator of the cell development process, the DNA repair system also participates in the innate antiviral response.

The transcriptome data were verified by qRT-PCR, and the criteria for gene selection were a high expression level, a large difference in expression, and Padj < 0.05. Additionally, in this study, we mainly focused on genes related to immune activation and infection enhancement, so we mostly selected some upregulated genes for verification, but for the rationality of the verification, we also selected individual downregulated genes. The results showed that the RNA-Seq and qRT-PCR data were highly consistent. The expression trends of 12 of the selected 13 genes were consistent with transcriptome analysis, indicating that the RNA-Seq data had a certain degree of credibility. Among these genes, 8 genes showed statistically significant differences in expression at 4 dpi, and most of them showed a trend in which the expression difference gradually increased with an increase in the number of days of infection. This study provides basic information for in-depth study of the mechanism of C6/36 cells infected with DENV2 and can be used as a candidate gene to block virus replication for further research.

The endoplasmic reticulum chaperone BiP (BiP/GRP78), a member of the heat shock protein 70 (Hsp70) family, is the main protein that binds to nascent immunoglobulins. It plays a key role in the folding of ATP-dependent chaperone proteins [59,60], ensuring that cells are protected from denatured proteins and enhancing their anti-apoptotic effects when cells are under stress [61]. The replication and assembly of flaviviruses mostly occur in the endoplasmic reticulum (ER) membrane factory. Host cells may exhibit an unfolded protein response (UPR), which is responsible for handling unfolded protein accumulation and stimulating appropriate cellular responses [62]. In ZIKV infection, the UPR is regulated by downregulation of the expression of BiP/GRP78 to evade the host defense system, and UPR inducers have also been found to significantly inhibit ZIKV replication [63]. This induction of BiP/GRP78 by DENV2 is essential for the synthesis of viral proteins in mosquito cells but has no important effect on viral RNA replication [64]. In our study, BiP/GRP78 expression was upregulated after infection with DENV2, and this change was statistically significant at 4 dpi. BiP/GRP78 may be an important factor in maintaining ER homeostasis, which is necessary to improve the survival rate of infected cells, while ER homeostasis may be disrupted by viral infection [65]. This provides us with information to study the regulatory mechanism of the UPR caused by DENV2 infection and improves our understanding of the virus–vector interaction in mosquitoes. Moreover, the expression differences in endoplasmin-like proteins at 1, 2, and 4 dpi were statistically significant, and these proteins are mainly involved in the processing and transportation of secreted proteins. These proteins are key host factors in flavivirus maturation and replication in the ER lumen. As effective inhibitors of ER-associated degradation (ERAD), the compound bardoxolone methyl (CDDO-me) and the endoplasmin inhibitor PU-WS13 can inhibit DENV2 replication and cytopathic effects caused by DENV infection [66]. Further research on ER-related pathway inhibitors can provide new directions for the development of new types of broad-spectrum antiviral drugs.

In addition, there were six other genes that showed statistically significant differences in expression at 4 dpi, including protein lifeguard 1, RNA polymerase II transcriptional coactivator, cold shock domain-containing protein E1-like, endoribonuclease Dicer, cystathionine beta-synthase (CBS), and facilitated trehalose transporter Tret1 (Tret1). These 8 genes that were upregulated in infected C6/36 cells may play a key role in infection. Moreover, the specific mechanisms of these genes involved in DENV2 infection need to be further studied. Based on the same experimental conditions, we conducted an overlap analysis of the transcriptome of C6/36 and Aag2 cells, and strived to find common genes and pathways in response to DENV2 infection in the two mosquito vectors, and provided us with a broad spectrum of related genes and pathways involved in viral infections with different mosquito vectors. Among them, the expression differences of CBS and Tret1 in DENV2-infected Aag2 cells were statistically significant. CBS is involved in the biosynthesis of hydrogen sulfide (H_2_S) and participates in redox status, DNA methylation, protein modification, and cell energetics regulation [67]. Trehalose is the main hemolymph sugar in most insects, and the Tret1 gene is involved in the transport of trehalose synthesized in fat bodies to hemolymph [68]. However, the expression pattern was opposite to that of DENV2-infected C6/36 cells, with the expression of the gene appearing to be downregulated at 4 dpi, indicating that these two genes were involved in the infection process via two pathways in C6/36 and Aag2 cells. The analysis revealed that some of the pathways involved in DENV2 infection of Aag2 and C6/36 cells are the same. This indicates that DENV2 infection can cause similar biological responses in different types of mosquitoes, which indicates that part of its infection mechanism in mosquitoes is conserved.

## 5. Conclusions

In this study, 1239 DEGs were screened by comparing the transcriptome data of infected and uninfected C6/36 cells, among which 1133 were upregulated and 106 were downregulated. Further bioinformatics analysis showed that upregulated DEGs were mainly enriched in signaling pathways such as the MAPK, Hippo, FoxO, Wnt, mTOR, and Notch pathways; metabolic pathways; and cellular physiological processes such as autophagy, endocytosis, and apoptosis. Downregulated DEGs were mainly enriched in DNA replication, pyrimidine metabolism, and repair pathways, including BER, NER, and MMR. Furthermore, we identified 8 significantly different genes in the qRT-PCR verification experiment, of which CBS and Tret1 were also differentially expressed in Aag2 cells. This research first studied the changes in the transcriptome level of *Aedes albopictus* C6/36 cell line infected with DENV2. The effect of DENV2 infection on mosquito is poorly known. It is very important to understand the mosquito vector–virus interaction, including antiviral response, immune evasion strategies for pathogen recognition, etc. At the same time, the application of transcriptome sequencing provides a faster and more effective method for mining the genes related to the susceptibility of mosquito-borne pathogens. The results of this study provide a lot of information about DENV2 infection in C6/36 cells at the transcriptome level. The data analysis results of this study provide a large number of *Aedes albopictus* virus infection-related regulatory genes and candidate antiviral genes, and provide basic data and directions for further research on the complex mechanism of vector–pathogen interactions. The analysis of these related genes and pathways provides new ideas and strategies for regulating or even blocking the replication and transmission of viruses in mosquito vectors.

## Figures and Tables

**Table 1:** Primer sequences of selected mRNA transcripts and the reference gene.

Gene	Gene Description	Primer	Length (bp)
LOC109405344	β-actin	F:GGAGAAGATCTGGCATCACA	95
		R:TGTCATCTTCTCGCGGTTAG	
LOC109413675	endoplasmic reticulum chaperone BiP	F:TCGAGAGCTACGCCTACAGT	97
		R:GCTTCCTCCATCTTGGCCTT	
LOC109396991	endoplasmin-like	F:GTACTCCATCTCCGCTCTGC	87
		R:TCCTCGGACTCGTTCAGGAT	
LOC109622167	protein lifeguard 1	F:GTGGAGAGATGCGACGGAAA	129
		R:TACCAACCGCCTTGAGAACC	
LOC109413087	RNA polymerase II transcriptional coactivator	F:GACTCCGCTAGCACAACCAA	140
		R:GGCAGGGATTGACCGTCTTT	
LOC115253702	mitochondrial carrier protein Rim2-like	F:AGTAACATGCCCGCTGGAAG	92
		R:TCGATTGGGCAATACGAGGC	
LOC109413857	cold shock domain-containing protein E1-like	F:ACGTTTTTGGTGGAGGTGACA	105
		R:CTCCACCGGACATGCTCATC	
LOC109403945	endoribonuclease Dicer	F:GGTGGATCCGAAGACTAGCG	114
		R:GTCTTGTCCCCCATGGTCTG	
LOC109426385	insulin-like growth factor 2 mRNA-binding protein 1	F:GTTCGAGAGCCACGAACAAG	108
		R:CGGTTCGCTTTGTTACTGTCG	
LOC109417697	glutathione S-transferase 1-like	F:CTTCTCGAGCCGTGGATCTC	127
		R:GGGATTGTATGCCGAGGGTT	
LOC109414692	protein kibra	F:TCACATCGTCAGCCGATTCC	107
		R:TCGTAAACAGCGACTGCGAT	
LOC109429036	UDP-glucuronosyl-transferase 2B1-like	F:ACGAGTCGGTCCAAGGTCTA	129
		R:CTCCCTCCTGAGTTCCCAGA	
LOC109427518	cystathionine beta-synthase	F:GCCACGTCCCAAGGTTATGA	101
		R:CATTGCACTTCAACCCTGCC	
LOC109409093	facilitated trehalose transporter Tret1	F:TCTTCGGGGGATGCTTTGTG	124
		R:CACCGGAAGGCATGCAGATA	

**Table 2:** The expression levels of 13 genes at 1, 2, and 4 dpi.

Gene	RNA-Seq		1 dpi		2 dpi		4 dpi	
	FC	Padj	FC	*p*	FC	*p*	FC	*p*
LOC109413675	2.08	0.000	1.19	0.275	1.02	0.711	4.49	0.000
LOC109396991	2.43	0.000	1.89	0.002	1.31	0.001	6.41	0.000
LOC109622167	3.30	0.000	1.04	0.801	0.96	0.667	1.74	0.002
LOC109413087	16.12	0.007	1.06	0.788	1.07	0.514	1.81	0.008
LOC115253702	4.10	0.009	0.69	0.360	0.72	0.057	1.62	0.097
LOC109413857	4.43	0.005	0.79	0.568	1.09	0.549	1.66	0.036
LOC109403945	7.04	0.000	0.86	0.701	0.96	0.648	3.26	0.004
LOC109426385	4.90	0.030	0.75	0.077	0.55	0.042	1.02	0.939
LOC109417697	0.29	0.041	0.84	0.554	0.74	0.035	0.84	0.313
LOC109414692	26.23	0.001	0.03	0.239	0.85	0.677	1.20	0.729
LOC109429036	0.55	0.015	2.65	0.008	1.85	0.003	1.23	0.487
LOC109427518	1.74	0.028	0.83	0.634	0.62	0.019	1.50	0.010
LOC109409093	1.40	0.050	0.95	0.887	1.07	0.638	2.07	0.001

## Data Availability

The datasets supporting the conclusions of this article are included within the article and its additional files. The raw transcriptome sequencing data are publicly available in the NCBI Sequence Read Archive (SRA) under BioProject ID PRJNA703391.

## Supplementary Materials

The following are available online at https://www.mdpi.com/1999-4915/13/2/343/s1. Table S1: List of differentially expressed genes. Table S2: GO enrichment analysis of upregulated differentially expressed genes. Table S3: GO enrichment analysis of downregulated differentially expressed genes. Table S4: KEGG pathway analysis of upregulated differentially expressed genes. Table S5: KEGG pathway analysis of downregulated differentially expressed genes.
